# Current Research and Development of Chemotherapeutic Agents for Melanoma

**DOI:** 10.3390/cancers2020397

**Published:** 2010-04-09

**Authors:** Kyaw Minn Hsan, Chun-Chieh Chen, Lie-Fen Shyur

**Affiliations:** Agricultural Biotechnology Research Center, Academia Sinica, Taipei 115, Taiwan, China; E-Mail: robin@gate.sinica.edu.tw (K.M.H.); maraca@gate.sinica.edu.tw (C.-C.C.)

**Keywords:** cutaneous malignant melanoma, metastasis, chemotherapeutic drugs, herbal medicines, phytoagents

## Abstract

Cutaneous malignant melanoma is the most lethal form of skin cancer and an increasingly common disease worldwide. It remains one of the most treatment-refractory malignancies. The current treatment options for patients with metastatic melanoma are limited and in most cases non-curative. This review focuses on conventional chemotherapeutic drugs for melanoma treatment, by a single or combinational agent approach, but also summarizes some potential novel phytoagents discovered from dietary vegetables or traditional herbal medicines as alternative options or future medicine for melanoma prevention. We explore the mode of actions of these natural phytoagents against metastatic melanoma.

## 1. Introduction to Melanoma

Skin cancer is one of the most common types of cancer, and cases are increasing yearly worldwide. In the United States, the estimated number of new skin-cancer cases in 2000 was 56,900 and this number grew by 31.12% in 10 years to 74,610 cases in 2009 [1,2]. The three main types of skin cancer are: (1) basal cell carcinoma formed in small cells in the base of the outer layer of the epidermis; (2) squamous cell carcinoma arising from the uppermost layer of the skin, and (3) melanoma, which begins in melanocytes, the cells that synthesize pigment in the skin [3]. Melanoma is the most aggressive and is also lethal. Risk factors of melanoma include family history, a previous melanoma incidence, gene polymorphisms, multiple moles, sun sensitivity, immune suppression, alcohol consumption, and exposure to ultraviolet radiation (UV). Many studies have examined the association of genetic polymorphisms with melanoma risk; for instance, variants of the DNA repair-related genes XPD/ERCC2 are associated with cutaneous melanoma [4]. Mutations of cyclin-dependent kinase (CDK) inhibitor 2A and 4 genes might be associated with family history of melanoma [5]. UV from sunlight induces DNA damage or suppresses the immune system of the skin, thus resulting in skin disorders, including melanoma [6]. Epidemiological studies have demonstrated heavy alcohol drinking associated with increased risk of melanoma [7].

The Clark model describes five events in the progression of normal melanocytes to malignancy: the formation of benign nevi from normal melanocytes, development into dysplastic nevi because of genetic lesions, radial growth phase, vertical growth phase and metastatic melanoma [8]. Several genes involved in the development of melanoma, including microphthalmia-associated transcription factor (MITF), c-Kit, V-raf murine sarcoma viral oncogene homolog B1 (BRAF) and neuroblastoma RAS viral oncogene homolog (N-RAS). MITF controls the development and function of melanocytes by regulating the expression of tyrosinase [9]. Both the overexpression and oncogenic role of MITF in melanoma development and progression has been demonstrated [10]. C-Kit has been linked to the promotion of cellular migration and proliferation of melanocytes. C-Kit is highly expressed in the early stage of melanoma but not in the late stage [11]. BRAF is a proto-oncogene that belongs to the serine/theronine kinase family. N-RAS is involved in mitogen-activated protein kinase (MAPK) signal transduction pathways that induce proliferation, survival and invasion of cells. Oncogenic BRAF and N-RAS mutations are frequent in malignant melanoma [12]. Nearly 60% of BRAF and 30% of N-RAS are mutated in melanomas. These mutations enhance proliferation and survival through stimulation of the MEK1/2-ERK1/2 mitogen-activated protein kinase pathway [13]. 

Treatment of melanoma can be efficient in the early stages; however, survival rates for malignant melanoma are low. The probability of survival for stage I and stage V melanoma is nearly 95% and less than 10%, respectively, five years after diagnosis [14]. After three decades of clinical trials of metastatic melanoma, new and effective agents are still needed for prolonging patient survival. Historically, objective response rate (tumor shrinkage) was used as a benchmark for the outcome phase II trials; however, in patients with metastatic stage IV melanoma, the agents conferred a promising objective response rate had not received meaningful effect on survival. Korn *et al*. developed alternative benchmarks−overall survival (OS) and progression-free survival (PFS) − for future phase II trials of melanoma aiming to identify appropriate agents worthy of phase III trials [15]. The proposed benchmarks were identified by meta-analysis of data collected from 2100 patients from 70 arms of 42 Southwest Oncology Group trials performed over 30 years (1975 to 2005). The authors proposed that the decision to advance a therapeutic intervention to a phase III trial depends on the difference in a trial’s benchmark between historical 1-year OS and observed 1-year OS rate defined by the distribution of prognostic factors (performance status, presence of visceral metastasis, sex, and exclusion of patients with brain metastasis) for the patients in the phase II trial. Six-month PFS rates are recommended for an early assessment of the agent in the trial, with 1-year OS used as the primary endpoint. The study of Korn *et al*. provides important, alternative benchmarks for identifying a new generation of therapies for phase III testing [16].

Conventional treatments for cutaneous melanoma include surgery, radiation therapy, immunotherapy, and chemotherapy. To enhance response rates in patients with metastatic melanoma, new approaches to treatment being evaluated in clinical trials include gene therapy, vaccine therapy, and targeted and combination therapy. In this article, we review current melanoma treatment with chemotherapeutic agents, targeted or combination therapy, or with phytocompounds and phytomedicines as novel or alternative approaches. 

## 2. Current Chemotherapeutic Agents

Chemotherapy involves the use of chemotherapeutic drugs to kill cancer cells or prevent them from growing. Current chemotherapeutic agents for cutaneous melanoma include alkylating agents, platinum drugs, and microtubule-toxin agents.

### 2.1. Alkylating Agents

Dacarbazine (DTIC) is the most commonly used alkylating chemotherapeutic agent and the first US Food and Drug Administration (FDA)-approved drug for the treatment of metastatic melanoma. DTIC interacts with nucleic acid molecules by covalent bonding and causes cytotoxic effects in cancer cells. The overall response rate is 15% to 20%, the median response duration is only 4 to 6 months, and complete response is less than 5% [17,18]. The main side effects are nausea, vomiting, bone marrow suppression, fever, aches, malaise and orthostatic hypotension [19]. In contrast to DTIC, orally available temozolomide (TMZ), an analog of DTIC, can penetrate the central nervous system; thus, TMZ is usually used to treat melanoma brain metastasis. TMZ used as a single agent in a phase II study had a response of 21%, a median response duration of approximately 5.5 months and 5% complete response [20]. In a phase III clinical trial of TMZ *versus* DTIC in the treatment of advanced metastatic malignant melanoma, TMZ and DTIC were equal in overall response rate, median response duration, complete response and toxicity profiles [21]. The main side effects of TMZ are constipation, fatigue, nausea, vomiting and low blood cell counts.

Nitrosoureas, another type of alkylating agent, including carmustine, lomustine, semustine, cystemustine and fotemustine, can cross the blood–brain barrier because of their lipophilic characteristics. Among them, cystemustine was the last to be synthesized and produced an overall response rate of 18% as a single agent [22]. Fotemustine was the first drug to show significant efficacy in brain metastasis [23]. A French multicenter phase II study demonstrated an overall response of 24%, a median response duration of 22 weeks, and 2% complete response with fotemustine [24]. The main side effects of nitrosoureas are thrombocytopenia, neutropenia, nausea and vomiting.

### 2.2. Platinum Drugs

The platinum compounds cisplatin, carboplatin, and zeniplatin inhibit DNA replication or transcription by triggering cross-linking of DNA molecules, which leads to DNA conformational changes. These compounds conferred modest activity in patients with metastatic melanoma. Cisplatin as a single agent produced an overall response of 15% and median duration of three months [25]. In another study, 150 mg/m^2^ cisplatin combined with amifostine (WR-2721) gave an overall response of 53% with median duration four months [26]. A phase II trial of carboplatin for advanced malignant melanoma showed a response rate of 19% with 7% complete response [27]. Zeniplatin is a platinum analogue, and a phase II study of zeniplatin in metastatic melanoma revealed an overall response rate of 14% [28]. The main side effects of platinum compounds are nephrotoxicity, neurotoxicity, ototoxicity, vomiting, and myelosuppression.

### 2.3. Microtubule-toxin Agents

Microtubule-toxin agents include taxanes and vinca alkaloids. These compounds can bind with tubulin and thus prevent the formation of microtubules [29]. Microtubules are responsible for cellular integrity and chromosome separation during mitosis. Thus, taxanes and vinca alkaloids maintain proliferation of cancer cells by triggering metaphase arrest. Taxanes, including paclitaxel and docetaxel, are extracted from the needles of *Taxus brevifolia* yew plants. Docetaxel has a response rate similar to that of paclitaxel, and a phase II study of paclitaxel as a single agent demonstrated a response rate of 16% with metastatic melanoma [30]. The main side effects of taxanes are neutropenia, oedema and alopecia. Vinca alkaloids include vincristine, vindesine and vinblastine. Among them, vindesine used as single agent showed an overall response rate of 19% [31]. The main side effects of vinca alkaloids are peripheral neuropathy and myelosuppression.

## 3. Targeted or Combination Therapy

A number of studies have indicated different signal pathways and anti-apoptotic proteins involved in survival of cancer cells. Thus, targeted drugs or small molecules serve as inhibitors of signal transduction pathways or as pro-apoptotic proteins to kill cancer cells or prevent them from proliferation [32]. Combination therapies were studied in the 1980s to improve response rates or overall survival in advanced melanoma; examples include different combinations of single-agent chemotherapeutic drugs and a combination of immunotherapeutic or targeted agents with chemotherapeutic chemicals. Here, we describe some examples of targeted or combination therapy.

### 3.1. Anti-apoptotic Protein Inhibitors

Anti-apoptotic proteins such as Bcl-2 are found in many cancers, including breast, colon, prostate, and lung carcinomas, as well as melanoma. Overexpression of Bcl-2 contributes to metastasis in melanoma. Thus, drugs such as oblimersen to downregulate Bcl-2 expression have been evaluated. Oblimersen is an antisense oligonucleotide that binds to the first 6 codons of Bcl-2 mRNA for degradation by RNase H. Hence, oblimersen acts as an anticancer agent by decreasing the level of Bcl-2 protein [33,34]. A phase III clinical trial of oblimersen combined with DTIC compared to DTIC alone indicated improved overall response rates (13.5% *vs.* 7.5%) but no significant improvement in overall survival. The main side effects of oblimersen include nausea, vomiting, fatigue, thrombocytopenia, pyrexia, anorexia and neutropenia [35].

### 3.2. Signaling Pathway Inhibitor

Phosphatidylinositol 3 kinase (PI3K)/Akt/mammalian target of rapamycin (mTOR) is an important pathway inducing the transformation and growth of melanoma. Everolimus, an oral drug, is a derivative of rapamycin and functions as an mTOR inhibitor to prevent rejection of organ transplants. Several studies indicated that everolimus was well tolerated and showed little dose-limiting toxicity in solid tumors [36,37]. The Ras/Raf/MAPK signaling pathway plays an important role in cell survival and proliferation and also mediates the activation of nuclear factor κB (NF-κB) to enhance metastasis and angiogenesis of melanoma [38]. Sorafenib, a multikinase inhibitor, is an FDA-approved agent for treating advanced primary renal cell carcinoma and advanced primary hepatocellular carcinoma. This multikinase inhibitor blocks Ras/Raf/MAPK signaling pathways by targeting B-Raf and C-Raf. It also inhibits vascular endothelial growth factor receptor (VEGFR)-1, -2 and -3 and autophosphorylation of platelet-derived growth factor receptor (PDGFR)-β tyrosine kinase to suppress angiogenesis. Sorafenib used as a single agent to treat melanoma had only modest activity: 19% response for stable disease. However, when combined with a chemotherapy agent, the drug showed more encouraging results [39]. The combination of sorafenib with paclitaxel and carboplatin in a phase I/II trial gave a high partial response rate (40%) and a 43% response for stable disease. However, the subsequent phase III trial found only 12% partial response and 54% response for stable disease [40]. Combining sorafenib with DTIC gave a 17% partial response and 61% response for stable disease in a phase I trial. A later double-blind randomized phase II trial found 24% partial response and 47% response for stable disease [41]. These data reveal better outcomes with DTIC combined with other agents than with FDA-approved DTIC alone for treating melanoma. 

PLX4032 and PLX4720 are available oral anti-cancer drugs developed by Plexxikon. They specifically target BRAF cancer carrying a mutation at V600E (designated V600E BRAF) that occurs in about 50%–60% of melanomas and about 6%–8% of all solid tumors, including colorectal, thyroid, and ovarian tumors [42]. PLX4720 inhibits V600E BRAF melanoma cells, with an IC_50_ of 13 nM, accompanied by potent inhibition of phosphorylation of ERK, which leads to cell cycle arrest and apoptosis. In a V600E BRAF xenograft mouse model, orally administered PLX4720 causes growth delay and regression of tumors [43]. A phase I trial of patients with metastatic melanoma, thyroid, rectal, or ovarian carcinoma receiving PLX4032 showed tumor regression in five of seven (83%) patients with V600E BRAF and two of four patients (50%) with unknown V600E status [44]. These seven patients with tumor regression remained free of tumor progression for 4 to 14 months, as compared with two patients with BRAF wild-type who showed progressive disease. These observations suggest the novel antitumor activity of PLX4032 against tumors with V600E BRAF mutations. Subsequent phase II and III trials of PLX4032 are under way.

Imatinib mesylate (Gleevec^TM^, Novartis), is a selective and competitive inhibitor of various tyrosine kinases such as KIT, BCR-ABL, ABL and platelet-derived growth factor receptors (PDGF-Rs) [45]. For instance, Gleevec binds to the active site of the mutationally active tyrosine kinase KIT and inhibits the protein, thereby stopping cellular signaling and turning off tumor growth. Two phase II trials evaluating the effects of imatinib in metastatic melanoma revealed a lack of clinical efficacy of the drug because of its high toxicity and low median overall survival and median time to progression [46,47].

Inhibitors of heat shock protein 90 bind to the ATP pocket in the N-terminal domain of the HSP90 molecular chaperone. Inhibition of the ATPase activity results in depletion of HSP90 client proteins, including c-RAF-1, Akt and CDK4. HSP90 chaperone is associated with BRAF mutational status. Evaluation of the anti-melanoma effects of 17-AGG tanespimycin, an inhibitor of HSP90, in a phase II trial revealed no anti-melanoma response [48].

### 3.3. Angiogenesis Inhibitor

Bevacizumab (Avastin) is a humanized monoclonal antibody that stops tumor growth by preventing the formation of new blood vessels through targeting and inhibiting the pro-angiogenic function of VEGF. A study of 12 patients who had previously received treatment for metastatic melanoma evaluated the combination of weekly administered paclitaxel and bevacizumab. Two patients (16.6%) showed a partial response and 7 (58.3%) stable disease. Median disease-free and overall survival times were 3.7 and 7.8 months, respectively [49]. A phase II trial of carboplatin combined with weekly paclitaxel and biweekly bevacizumab enrolled 53 patients with unresectable stage IV melanoma. Nine (17%) patients showed partial remission and another 30 (57%) stable disease for at least eight weeks. Median progression-free and overall survival was 6 and 12 months, respectively [50]. The main side effects were neutropenia, thrombocytopenia, hypertension and anemia. Thus, the angiogenesis inhibitor bevacizumab appears to be moderately well tolerated and clinically beneficial for patients with metastatic melanoma.

### 3.4. Combination of Double Chemotherapeutic Agents

Evaluation of the combination of *cis*-platinum and DTIC chemotherapy demonstrated a 30% response rate, median response duration of 31 weeks and 7% complete response [51]. This response rate was better than in a previous experiment with the same combination therapy. The combination of 2 chemotherapeutic agents, dacarbazine and thalidomide, in a phase II trial for the treatment of metastatic melanoma evaluated in 15 patients with stage IV disease conferred inefficient outcomes and high toxicity [52]. Of 30 patients with metastatic cutaneous melanoma who received combined treatment with temozolomide and cisplatin, 16.7% showed partial response, 20% stable disease and 56.6% progressive disease [53]. 

### 3.5. Combination of 3 Chemotherapeutic Agents

In a clinical trial of the combination of three drugs—docetaxel, temozolomide and cisplatin—for advanced melanoma, of 23 patients enrolled, 32% showed partial response and 26% stable disease, which was higher than with treatment with the single agents, so this combination seemed to be promising treatment for malignant melanoma [54]. However, the main side effects of this regimen were myelosuppression and pulmonary embolism. In a recent trial of 46 patients who received treatment with the triple combination of cisplatin, taxol and dacarbazine, two patients showed complete response and 17 partial response, with an overall response rate of 41%. Side effects included myelosuppression and neuropathy. The results suggested that cisplatin-paclitaxel-dacarbazine treatment is an effective regimen for patients with metastatic melanoma [55].

### 3.6. Combination of Chemotherapeutic and Immunotherapeutic Agents (Biochemotherapy)

A randomized study of 400 patients receiving treatment with a combination of the chemotherapeutic agents cisplastin, vinblastine, DTIC (CVD) or a combination of CVD with interleukin 2 (IL-2) and interferon α (IFN-α) (biochemotherapy) revealed a response rate of 11% and 17% for the CVD and biochemotherapy arms, respectively. The two arms did not differ in complete response or overall survival [56]. A recent phase II biochemotherapy trial estimating the efficacy and tolerability of fotemustine, cisplatin, IL-2 and IFN-α for advanced melanoma evaluated 60 patients, including 12 with brain metastasis. One patient showed complete response and 10, including one with brain metastasis, showed partial response. The overall response rate was 18.4% and was 16.7% for patients with brain metastasis. The outcomes also indicated a high percentage of stable disease: 58.4% for all patients and 75% for patients with brain metastasis [57]. Although this combination showed encouraging results, especially for patients with brain metastasis, subsequent clinical trials are needed to confirm the effects of this regimen.

## 4. Alternative Phytomedicine Treatment of Melanoma

Several studies demonstrated that chemotherapeutic agents or combination therapies had many side effects and low response rate in the treatment of melanoma. In recent years, identification and validation of the potential benefits of phytocompounds has become an important area of pharmaceutical science. Natural compounds may be considered alternative means for melanoma prevention because of low or little toxicity due to their dietary properties or their long history as herbal medicines. Several recent studies examined the use of natural compounds for treating melanoma as targeted treatment or their efficacy in combination with clinical chemotherapeutic drugs for cutaneous melanoma. Some examples of well-known phytocompounds from dietary vegetables or traditional herbal medicines are summarized in Table 1 and also described below.

**Table 1:** Natural phytocompounds and herbal medicines in the treatment of cutaneous melanoma.

Phytochemicals	Mode of actions/targets	References
*Phenolics*		
catechins (-)-epigallocatechin-3-gallate (EGCG) (single or in combination with IFN α-2b or dacarbazine)	anti-proliferative and proapoptotic effects Fas/FasL signaling pathway, Bax, caspases-3, -7 and -9 ↑ tumor growth, metastasis, cell spreading, cell-extracellular matrix, MMP-9, FAK ,NF-κB pathway, and Bcl-2 ↓	[58,59,60,61,62,72]
resveratrol	NQO2 and p53 ↑; cell proliferation ↓	[65,66]
piceatannol	arrest at cell cycle G_2_ phase; cyclins A, E, and B1 ↓	[67]
curcumin	anti-proliferative and proapoptotic effects; caspase-3 ↑ tumor growth, metastasis, NF-κB, COX-2, MMP, and PRL-3 ↓	[69,70,71,72]
apigenin, quercetin, and kaempferol	Bcl-2, p27, and p21 ↑ tumor growth, invasion, metastasis, CDK1, and CDK2 ↓	[73,74]
genistein plus cisplatin	Apaf-1 ↑; Bcl-2 and Bcl-xL ↓	[77]
*Alkaloids*		
punarnavine	immune response, IL-2, and IFN-γ ↑ metastasis, IL-1β, IL-6, TNF-α, MMP-2, MMP-9, ERK-1, ERK-2, and VEGF ↓	[78,79]
9-carbethoxy-3-methylcarbazole, 9-formyl-3-methylcarbazole 1-*O*-acetylnorpluviine, sternbergine 1-epideacetylbowdensine, crinamine, crinine, hamayne, lycorine, anhydrolycorin-6-one	cytotoxic to melanoma cells	[80,81]
matrine	anti-proliferative and apoptotic effects; invasion, metastasis, and heparanase ↓	[107]
ligusticum	metastasis, CD34, and VEGF ↓	[108]
berberine	anti-proliferative and necrotic effects tumor growth ↓	[109]
*Terpenoids*		
13-*cis*-retinoic acid (13-*cis*-RA), all-*trans*-retinoic acid (ATRA) (or in combination with cisplatin and 5-fluorouracil)	anti-proliferative and anti-invasive effects lung metastatic melanoma ↓	[83]
β-carotene	caspases-3, -8 and -9 , cytochrome c release, and t-BID ↑; Bcl-2 ↓	[84]
lycopene	PDGF-BB and cell migration of stromal fibroblasts ↓	[85]
ginsenoside-Rh2 (G-Rh2)	apoptotic effect; caspase-3 and caspase-8 pathway ↑	[105]
oridonin	anti-proliferative and apoptotic effects EGFR, Grb2, Ras, and Raf-1 ↓	[110,111]
triptolide	tumor growth and cell proliferation ↓	[112]
*Galactolipid* *& polyunsaturated fatty acids*		
1,2-di-*O*-α-linolenoyl-3-*O*-β-galactopyranosyl-*sn*-glycerol (dLGG)	tumor growth, NF-κB, NO, iNOS, COX-2, and PGE_2_ ↓	[92]
n-3 PUFAs	PTEN pathway ↑; angiogenesis and metastasis ↓	[95,96]
*Plant hormone*		
jasmonic acid (JA)	tumor growth and metastasis ↓	[99]
methyl jasmonate (MJ)	apoptotic effect; caspase-3 ↑	
*Xanthone*		
gambogic acid	anti-proliferative effect; Bax/Bcl-2 ratio and caspase-3 ↑	[106]
*Isothiocyanates*		
sulforaphane (SFN)	apoptotic effect; IL-2, and IFN-γ ↑ tumor growth, invasion, metastasis, MMP, IL-1β, IL-6, TNF-α, and GM-CSF ↓	[101,102]
*Vitamins*		
γ-tocotrienol (γ-T3) (single or in combination with docetaxel and dacarbazine)	anti-proliferative and anti-invasive effects E-cadherin, γ-catenin, caspases-3, -7 and -9 ↑ Id-1, Id-2, EGFR, NF-κB p65, Snail, twist, α-SMA, and vimentin ↓	[103]
vitamin C (ascorbic acid)	p38 MAPK, IGF II, IGF-IR, and COX-2 ↓	[104]

↑: up-regulation or activation; ↓: down-regulation or inhibition.

### 4.1. Phenolic Compounds

Several phenolic compounds were tested for their inhibition of melanoma *in vitro* and *in vivo*, and results suggested a possible use of these compounds in arresting the metastatic growth of tumor cells.

#### 4.1.1. Catechins and (-)-epigallocatechin-3-gallate (EGCG)

Green tea catechins possess anti-tumorgeneic activity against various cancers, including melanoma [58,59]. Nihal *et al.* found EGCG, one of the tea catechins, with anti-proliferative and proapoptotic effects on melanoma cells but no influence on normal melanocytes at the same tested concentration [60]. Furthermore, EGCG could sensitize human melanoma cells to IFN-α2b induced tumor growth inhibition in a mouse model by activating Fas signaling and inhibiting the NF-κB pathway [61]. EGCG might have a therapeutic advantage by reducing toxicity and increasing the curative effect of IFN. A combination of EGCG and dacarbazine treatment lowered the threshold effective dose of dacarbazine and increased the survival rate of tumor-bearing mice by strongly inhibiting lung metastasis of B16-F3m melanoma [62]. These results suggested that single EGCG treatment or its combination with other drugs might be more effective in inhibiting melanoma growth and metastasis.

#### 4.1.2. Resveratrol and Piceatannol (3,5,3',4'-tetrahydroxy-*trans*-stilbene; PICE)

Found in fruits, roots, and pines, resveratrol is an anti-inflammatory phytocompound with therapeutic potential. It has numerous effects on several cancers [63]. Jang *et al.* demonstrated that resveratrol could prevent skin cancer development in a two-stage carcinogen-induced mouse model [64]. Resveratrol inhibiting melanoma cell proliferation is associated with up-regulation of tumour suppressor gene p53 and quinone reductase 2 activity [65]. Resveratrol also markedly impaired the proliferation of both TMZ-sensitive M14 and TMZ-resistant SK-Mel-28 or PR-Mel cell lines [66]. As well, the resveratrol analogue piceatannol caused cell-cycle arrest at the G_2_ phase by down-regulating the expression of cyclins A, E and B1 [67].

#### 4.1.3. Curcumin

Curcumin is a non-steroidal polyphenol existing extensively in the ginger family. Treatment with curcumin increased the expression of c-Jun and c-Fos to protect the skin against harm, so it was suggested as a potent inhibitor of the initiation and promotion of UVB light-induced skin carcinogenesis in mouse model [68]. Curcumin inhibited lung metastasis induced by B16F-10 melanoma [69]. The molecular mechanism of the curcumin anti-proliferative and proapoptotic effects on melanoma cells was through down-regulating the activity of matrix metalloproteinase (MMP), NF-κB and the phosphatase of regenerating liver 3 (PRL-3) [70,71]. Treatment with curcumin or catechin inhibited lung metastasis, tumor formation, and B16F-10 melanoma cells passing through the collagen matrix by suppressing metalloproteinases [72].

#### 4.1.4. Flavonoids: Apigenin, Quercetin, and Kaempferol

Flavonoids in the phytonutrient family are known for high antioxidant activity. Antioxidants are renowned for removing cell-damaging free radicals from the body, thereby possibly reducing the symptoms of aging and the risks of chronic diseases such as cancer. Numerous studies have shown that flavonoids such as apigenin, quercetin, and kaempferol effectively suppress melanoma growth and metastasis by down-regulating Bcl-2 and CDK expression and promoting CDK inhibitors (p27 and p21) and Bax expression, thus leading to apoptosis of melanoma cells [73,74]. 

#### 4.1.5. Isoflavonoids: Genistein

Genistein is an isoflavonoid phytoestrogenic compound found in soybean, pea pods, and other legumes. It is a tyrosine-specific kinase inhibitor that blocks phosphorylation of tyrosine residue in histone H2B [75]. Genistein also prevented UV-induced DNA damage in human melanoma cells [76]. In addition, Tamura *et al.* demonstrated that genistein can enhance cisplatin sensitivity to treatment in several melanoma cell lines via down-regulated Bcl-2 and Bcl-xL expression, and up-regulated expression of apoptotic protease activating factor 1 (Apaf-1) [77].

### 4.2. Alkaloids

The alkaloid punarnavine is purified from *Boerhaavia diffusa* Linn, a creeping and spreading perennial herb distributed throughout India. Punarnavine suppressed or down-regulated the expression of proinflammatory cytokines (IL-1β, IL-6 and TNF-α), metastasis-related proteins (MMP-2, MMP-9, ERK-1, ERK-2), and angiogenesis-related VEGF in melanoma-bearing mice and melanoma cells [78,79]. As well, the alkaloids 9-carbethoxy-3-methylcarbazole and 9-formyl-3-methylcarbazole purified from *Murraya koenigii* [80] and the alkaloids 1-*O*-acetylnorpluviine, 1-epideacetyl-bowdensine, crinamine, crinine, hamayne, lycorine, anhydrolycorin-6-one and sternbergine extracted from *Brunsvigia radulosa* [81] showed anti-proliferative effects in melanoma cells.

### 4.3. Terpenoids

Taxol is a well-known diterpenoid compound used to treat many cancers. Another well-known diterpenoid is retinol (vitamin A). Vitamin A is a fat-soluble vitamin found in animal food such as fish oil and liver. The effect of retinol might be due to inhibition of tyrosinase activity in mouse B16 melanoma cells, and it is a potential natural nutrient for developing an anti-melanogenic agent [82]. Retinoids, derivatives of vitamin A, are becoming popular as treatment for skin disorders. A study has been evaluated *in vitro* and *in vivo* effects of different retinoids, including all-*trans*-retinoic acid (ATRA), 13-*cis*-retinoic acid (13-*cis*-RA), 9-*cis*-retinoic acid, acitretin, and etretinate on tumor development and metastasis of melanoma by Liu *et al*. The results indicated that only ATRA and 13-*cis*-RA showed potent anti-proliferative and anti-invasive effects on B16F-10 melanoma cells. Besides, the number of lung metastatic colonies in C57BL/6J mice was significantly reduced by injecting ATRA and 13-*cis*-RA. Furthermore, 13-*cis*-RA can enhance cisplatin sensitivity to reduce tumor size and metastatic incidence on melanoma-bearing mice [83].

Carotenoids belong to the tetraterpenoids, including lycopene, carotene, and others. β-Carotene is probably the most well known of the carotenoids, able to be converted in the body into retinol, an active form of vitamin A. β-Carotene can inhibit cell proliferation and migration in several cancers, including melanoma. A study demonstrated that β-carotene activated caspase cascade (capases-9, -8 and -3) in human SK-MEL-2 melanoma cell line through activation of NF-κB; moreover, a dose-dependent decrease in the anti-apoptotic protein Bcl-2 and increase in the cleaved form of BID and cytochrome c release were observed, leading to apoptosis in the cancer cells [84]. Platelet-derived growth factor (PDGF) is one of the growth factors that regulate melanoma growth and division and has a significant role in angiogenesis. Binding assay showed that lycopene can bind an homodimeric PDGF-BB protein *in vitro* and *in vivo* (plasma); furthermore, melanoma cell migration of stromal fibroblasts was inhibited [85]. 

A phase II evaluation of the combination of IFN-α2a and ATRA demonstrated a median survival of only eight months for 52 patients with stage IV melanoma. An objective response rate of 10% was seen in the 52 patients with two complete and three partial responses. Further studies of IFN-α2a and ATRA in advanced melanoma are not warranted [86]. In another phase II study, 22 patients with stage IV melanoma received treatment for five consecutive days with CVD, as well as low-dose IL-2, pegylated IFN-α2b, and oral 13-*cis*-RA during chemotherapy. This combined therapy significantly prolonged overall and progression-free survival [87]. Regarding epidemiological studies, a case-control study (final sample of 497 melanoma cases and 561 randomized controls) showed that diets high in fruits and vegetables enrich sources of carotenoids (α-carotene, β-carotene, cryptoxanthin, lutein, and lycopene), with adjusted odds ratios (ORs) ≤0.67, which may be associated with reduced risk of melanoma [88]. However, a large prospective cohort study found no association of use of supplemental β-carotene and melanoma risk [89].

### 4.4. Galactolipid and Polyunsaturated Fatty Acids (PUFAs)

#### 4.4.1. 1,2-Di-*O*-α-linolenoyl-3-*O*-ß-galactopyranosyl-sn-glycerol (dLGG)

Natural galactolipids are galactosyl glycerols etherified with two fatty acid moieties. Monogalactosyldiacylglycerol (MGDG) is a major lipid constituent in plastids, found in green vegetables, that showed inhibition of human umbilical vein endothelial cell tube formation and cell proliferation in several cancers [90,91]. Recently, a bioactive MGDG, namely dLGG, identified from a medicinal plant *Crassocephalum rabens* (Asteraceae), had a significant inhibitory effect on inflammatory mediators such as NF-κB, inducible nitric oxide synthase, cyclooxygenase-2 (COX-2), and prostaglandin E_2_ (PGE_2_) in lipopolysaccharide-stimulated RAW 264.7 cells. Intraperitoneal administration of dLGG significantly inhibited the growth of B16 melanoma in mice with an effect comparable to that of cisplatin [92]. This type of galactolipid has potential for further development as a chemopreventive agent for melanoma.

#### 4.4.2. n-3 PUFAs

A number of studies have demonstrated that supplementation with n-3 PUFAs had therapeutic benefits in various disorders [93]. The omega-3 fatty acids can prevent non-melanoma skin cancers [94] and melanoma by enhancing apoptosis and reducing angiogenesis; in turn, lung colonization was inhibited in melanoma-bearing animals [95]. *In vitro* study by addition of n-3 fatty acid eicosapentaenoic acid or PGE_3_, a metabolite of n-3 fatty acid, in B16 melanoma cell culture showed that the cancer cell growth was significantly inhibited along with the over-expression of phosphatase and tensin homologue deleted on the chromosome 10 (PTEN) protein. Furthermore, the balance of n-6/n-3 fatty acid in dietary with the proportion of n-3 PUFAs exceeded that of n-6 PUFAs was found with higher therapeutic benefit. Xia *et al*. reported that B16 melanoma growth was reduced in a fat-1 transgenic mice engineered with a n-3 fatty acid desaturase that catalyzes converting n-6 to n-3 fatty acids [96]. 

### 4.5. Plant Hormone: Jasmonate Family

The jasmonate family contains *cis*-jasmone, jasmonic acid (JA), and methyl jasmonate (MJ). JA is a plant stress hormone responsible for regulation of plant growth and development and is a newly identified natural compound with anti-cancer activity [97]. JA affected mitochondrial function in cancer cells but not normal cells; in addition, JA suppressed cell proliferation and induced cell-cycle arrest and death in cancer cells [98]. Other study showed that JA affected skin carcinogenesis and suppressed the growth and lung metastasis of melanoma [99]. MJ elevated apoptotic proteins such as caspase-3 to induce an extrinsic apoptotic signaling in melanoma cells [100]. 

### 4.6. Other Nutritional Agents

A high dietary intake of cruciferous vegetables has been associated with reduced incidence of melanoma and was demonstrated to be helpful in treating skin diseases such those resulting from rubbing or scratching. Sulforaphane (SFN), one of isothiocyanates found in cruciferous vegetables such as broccoli and cauliflower, is a phase 2 enzyme inducer (including glutathione transferase, epoxide hydrolase, quinone reductase, and glucuronosyltransferase), which can prevent normal cell mutation and cancer cell development. SFN inhibits melanoma growth, invasion, and metastasis by inducing apoptosis, decreasing metastasis-related enzyme (MMP), and modulating immune responses (up-regulation of IL-2 and IFN-γ and down-regulation of proinflammatory cytokines) [101,102]. Another nutritional agent, γ-tocotrienol (γ-T3), an unsaturated form of natural vitamin E present in rice bran and palm oil, was found to have anti-proliferative and anti-invasive effects in malignant melanoma by restoring E-cadherin and γ-catenin expression and suppressing that of mesenchymal markers such as Snail, twist, *α*-smooth muscle actin, and vimentin. Furthermore, treatment with γ-T3 alone or in combination with docetaxel and dacarbazine activates caspases and suppresses EGFR and NF-κB expression in malignant melanoma [103]. Vitamin C (ascorbic acid) was recently reported to suppress the proliferation of the human melanoma cell line SK-MEL2 via down-regulating insulin-like growth factor (IGF) II, type I IGF receptor (IGF-IR), and COX-2 and altering p-p38 expression [104].

### 4.7. Traditional Chinese Medicine

Traditional Chinese Medicine (TCM) has been practiced for thousands of years. However, in the past decade, great effort from scientists worldwide has contributed to the identification of active ingredients for a particular medical usage for several TCMs composed of single or multiple herbal components. Ginseng is one of the most commonly used and researched TCMs. It is often used orally as an adaptogen, aphrodisiac and nourishing stimulant. Red ginseng extracts or ginsenoside-Rh2 (G-Rh2) possess anti-proliferative activity by inducing caspase-3 and caspase-8−dependent apoptosis in melanoma cells [105]. Gamboge, another TCM from the gamboge tree (genus *Garcinia*), is effective in combating inflammation, clearing toxins and stopping bleeding. Gambogic acid, a major active ingredient of gamboges, inhibited the proliferation of human malignant melanoma A375 cells by regulating the ratio of Bax to Bcl-2 and inducing caspase-3 activity [106]. 

Matrine, a major alkaloid component found in the roots of Sophora plants, can be obtained primarily from *Sophora japonica* and *Sophora subprostrata*. The main clinical applications are treatment of cancer, viral hepatitis, cardiac diseases, and skin diseases (such as psoriasis and eczema). Matrine inhibits the invasiveness and metastasis of human A375 melanoma cells. As well, heparanase, an important enzyme in the degradation of the extracellular matrix, is down-regulated by matrine [107]. Berberine, a plant alkaloid, usually exists in the roots, rhizomes, stems, and bark of barberry (*Berberis vulgaris*), goldenseal (*Hydrastis canadensis*), and coptis or goldenthread (*Coptis chinensis*). Some clinical applications have indicated the curative effects of berberine, including protection against bacterial diarrhea, intestinal parasites, and ocular trachoma infections; increasing cardiovascular functions; and anti-inflammatory activity. Berberine causes cell apoptosis and necrosis of B16 melanoma cells [108].

*Chuanxiong*, commonly called ligusticum or cnidium, is a Chinese herb frequently used to improve blood circulation; for example, amenorrhea, dysmenorrhea and irregular menstruation are treated with the root of this plant. Ligustrazine, isolated from *Chuanxiong*, inhibits metastasis of nodi to the lung by suppressing CD34 and VEGF expression in melanoma [109]. Oridonin, also known as rubesecensin A, is a diterpenoid isolated from the Chinese medicinal herb *Rabdosia rubescens.* It has been used in China to treat swelling of the throat, insect and snake bites, inflammation of the tonsils, and several cancers [110]. Downstream genes of epidermal growth factor receptor (EGFR), including Grb2, Ras and Raf-1, have been found in highly proliferative tissues and melanoma cells. Previous study indicated that Oridonin induced human A431 melanoma cell apoptosis by suppressing EGFR signaling [111]. *Tripterygium wilfordii* Hook. f., has been used in Chinese medicine for centuries. A diterpenoid, triptolide, was first isolated from this plant and tested in the treatment of solid tumors, including melanoma. Triptolide at low concentrations (2–10 ng/mL) inhibited tumor cell proliferation and the growth of xenografted melanoma in mice [112].

## 5. Concluding Remarks

In conclusion, current treatment for metastatic melanoma is still poor in terms of overall response and survival rate. Although some biochemotherapy trials have shown good outcomes, no agent or regimen has improved median response duration and overall survival in subsequent studies. New treatment options, as single or in combination, with the use of natural phytocompounds or herbal medicines may provide breakthrough results in the treatment of malignant melanoma.

## Correction

Correction Correction (PDF, 65 KB)
