# Supplementary material for: Current Research and Development of Chemotherapeutic Agents for Melanoma
Source: Cancers (Basel). 2010 Apr 9;2(2):397–419. doi: 10.3390/cancers2020397 (PMC3835084; doi:10.3390/cancers2020397)
Supplement: Correction — (PDF, 65 KB) [file cancers-02-00397-s001.pdf]

Cancers **2010**, *2*, 397-419; doi:10.3390/cancers2020397

OPEN ACCESS

cancers

ISSN 2072-6694

www.mdpi.com/journal/cancers

Supplementary file

Shyur *et al.* Current Research and Development of Chemotherapeutic Agents for Melanoma

Lie-Fen Shyur

Agricultural Biotechnology Research Center, Academia Sinica, Taipei 115, Taiwan;
E-Mail: lfshyur@ccvax.sinica.edu.tw

We found a mistake at the affiliation in our paper published in *Cancers* [1]. The correct affiliation is as below.

Kyaw Minn Hsan [1,2,†], Chun-Chieh Chen [1,2,†], and Lie-Fen Shyur [2,*]

[1] Institute of Plant Biology, National Taiwan University, Taipei 106, Taiwan;
[2] Agricultural Biotechnology Research Center, Academia Sinica, Taipei 115, Taiwan;
E-Mail: robin@gate.sinica.edu.tw (K.M.H.); maraca@gate.sinica.edu.tw (C.C.C.)

† Both authors contributed equally to this article.

* Author to whom correspondence should be addressed; E-Mail: lfshyur@ccvax.sinica.edu.tw;
Tel.: +886-2-26515028.

Reference

1. Hsan, K.M.; Chen, C.-C.; Shyur, L.-F. Current Research and Development of Chemotherapeutic Agents for Melanoma. *Cancers* **2010**, *2*, 397–419.

© 2010 by the authors; licensee MDPI, Basel, Switzerland. This article is an open access article distributed under the terms and conditions of the Creative Commons Attribution license (http://creativecommons.org/licenses/by/3.0/).
